# Anti-HMGB1 mAb Therapy Reduces Epidural Hematoma Injury

**DOI:** 10.3390/ijms25115889

**Published:** 2024-05-28

**Authors:** Shangze Gao, Dengli Wang, Keyue Liu, Yasuko Tomono, Li Fu, Yuan Gao, Yohei Takahashi, Mariko Yata, Masahiro Nishibori

**Affiliations:** 1 Department of Translational Research & Drug Development, Okayama University Graduate School of Medicine, Dentistry and Pharmaceutical Sciences, Okayama 7008558, Japan; gaoshangze@mail.tsinghua.edu.cn (S.G.); yasuko-tomono2021@outlook.jp (Y.T.); l.fu@yale.edu (L.F.); goreeouan@gmail.com (Y.G.); tyohey@mw.kawasaki-m.ac.jp (Y.T.); yatam@okayama-u.ac.jp (M.Y.); 2 School of Pharmaceutical Sciences, Tsinghua University, Beijing 100082, China; 3 Department of Pharmacology, Okayama University Graduate School of Medicine, Dentistry and Pharmaceutical Sciences, Okayama 7008558, Japan; dengliwang@okayama-u.ac.jp (D.W.); liukeyue@md.okayama-u.ac.jp (K.L.); 4 Faculty of Health Science and Technology, Kawasaki University of Medical Welfare, Okayama 7010193, Japan

**Keywords:** epidural hematoma, HMGB1, inflammatory response

## Abstract

Epidural and subdural hematomas are commonly associated with traumatic brain injury. While surgical removal is the primary intervention for these hematomas, it is also critical to prevent and reduce complications such as post-traumatic epilepsy, which may result from inflammatory responses in the injured brain areas. In the present study, we observed that high mobility group box-1 (HMGB1) decreased in the injured brain area beneath the epidural hematoma (EDH) in rats, concurrent with elevated plasma levels of HMGB1. Anti-HMGB1 monoclonal antibody therapy strongly inhibited both HMGB1 release and the subsequent increase in plasma levels. Moreover, this treatment suppressed the up-regulation of inflammatory cytokines and related molecules such as interleukin-1-beta (IL-1β), tumor necrosis factor-alpha (TNF-α), and inducible nitric oxide synthase (iNOS) in the injured areas. Our in vitro experiments using SH-SY5Y demonstrated that hematoma components—thrombin, heme, and ferrous ion— prompted HMGB1 translocation from the nuclei to the cytoplasm, a process inhibited by the addition of the anti-HMGB1 mAb. These findings suggest that anti-HMGB1 mAb treatment not only inhibits HMGB1 translocation but also curtails inflammation in injured areas, thereby protecting the neural tissue. Thus, anti-HMGB1 mAb therapy could serve as a complementary therapy for an EDH before/after surgery.

## 1. Introduction

Traumatic brain injuries resulting from traffic accidents and falls frequently lead to cranial fractures, often accompanied by subdural and epidural hematomas (EDH) [1]. The hematomas not only cause direct compression injury of the brain but also induce neural cell damage through clot constituents [1]. Therefore, early neurosurgical intervention is critical to protect brain tissue from ischemia and heme/iron-induced toxicity.

Elderly people, who are prone to hematomas, even from minor impacts such as falls, may exhibit slow-growing hematomas that become significant before detection [2]. Neurological deficits arise both from the general decrease in consciousness level and from symptoms that are specific to the damaged brain areas. Furthermore, it is well-known that clot-derived constituents such as thrombin, heme, and iron facilitate inflammatory responses, which are implicated in the onset of post-traumatic epilepsy [3]. Given the established link between the inflammatory responses and both epileptogenesis and the formation of epileptic foci [4,5], coupled with the aggravation of inflammation and subsequent brain tissue damage by reactive oxygen species (ROS) generated from free heme/iron in hemoglobin [5], the timely removal of hematomas is essential to prevent post-traumatic epilepsy.

In neonates, both preterm birth and very low birth weight are significant risk factors for intracranial bleeding [6]. Hemorrhage during antenatal, perinatal, and neonatal periods, particularly when associated with ischemia/hypoxia, can severely affect ongoing brain network development, potentially impairing a wide range of higher nervous functions [7]. A bleeding tendency, such as hemophilia, constitutes another risk factor for intracranial hemorrhage [8]. However, the protective therapies against such situations remain limited.

High mobility group box-1 (HMGB1) is a non-histone DNA-binding protein that functions as a pivotal damage-associated molecular pattern (DAMP). It is involved in many inflammatory diseases by directly activating plural receptors, forming complexes with other cytokines, and impeding hemoglobin clearance from extracellular space. In the previous studies, we found that HMGB1 translocation in neurons was evident after ischemic insult in rat brains [9,10]. A similar pattern of HMGB1 translocation and subsequent extracellular release in neurons was also observed in conditions such as intracerebral hemorrhage, fluid percussion-induced traumatic injury, and drug-induced epilepsy [4,11,12]. The released HMGB1 then, in turn, facilitates the disruption of the blood-brain barrier (BBB) by inducing the contraction of vascular endothelial cells, activating microglia, and causing the swelling of astrocyte end-feet, which collectively leads to brain edema. In addition, the released HMGB1 may act synergistically with other DAMPs like peroxiredoxins (Prxs) and chaperon molecules heat shock proteins (HSPs) to enhance the inflammatory response [13]. The systemic administration of the anti-HMGB1 monoclonal antibody (mAb) in animal models of stroke, brain trauma, and epilepsy significantly inhibited the translocation and release of HMGB1 from neurons, thus protecting the BBB from disruption [4,9,10,11,12]. In addition, anti-HMGB1 treatment suppressed brain inflammation by inhibiting the expression of cytokines and inflammation-related molecules. Taken together, we speculated that HMGB1 release may act as an upstream event in a cascade leading to a diverse range of brain injuries.

In the present study, we aimed to evaluate the efficacy of anti-HMGB1 mAb therapy in mitigating EDH-induced brain inflammation and damage in rats. Specifically, we investigated whether the systemic injection of the anti-HMGB1 mAb could reduce the release of HMGB1 from the brain injury area and suppress inflammatory responses beneath the EDH. Additionally, we assessed brain edema by examining AQP4, a key water channel protein involved in brain edema, and PAR-1/PAI-1, regulators of fibrinolysis, as well as macrophage scavenger receptor 1 (MSR1), which is involved in the clearance of cellular debris. Furthermore, we examined the effects of the anti-HMGB1 mAb on HMGB1 release from both neuronal cells and vascular endothelial cells in vitro. Our goal was to explore the potential of anti-HMGB1 therapy as a novel and unique method for treating brain hemorrhage, independent of neurosurgical interventions.

## 2. Results

### 2.1. HMGB1 Level Changes in the Brain and Plasma 48 h after the EDH

**Figure 1:**
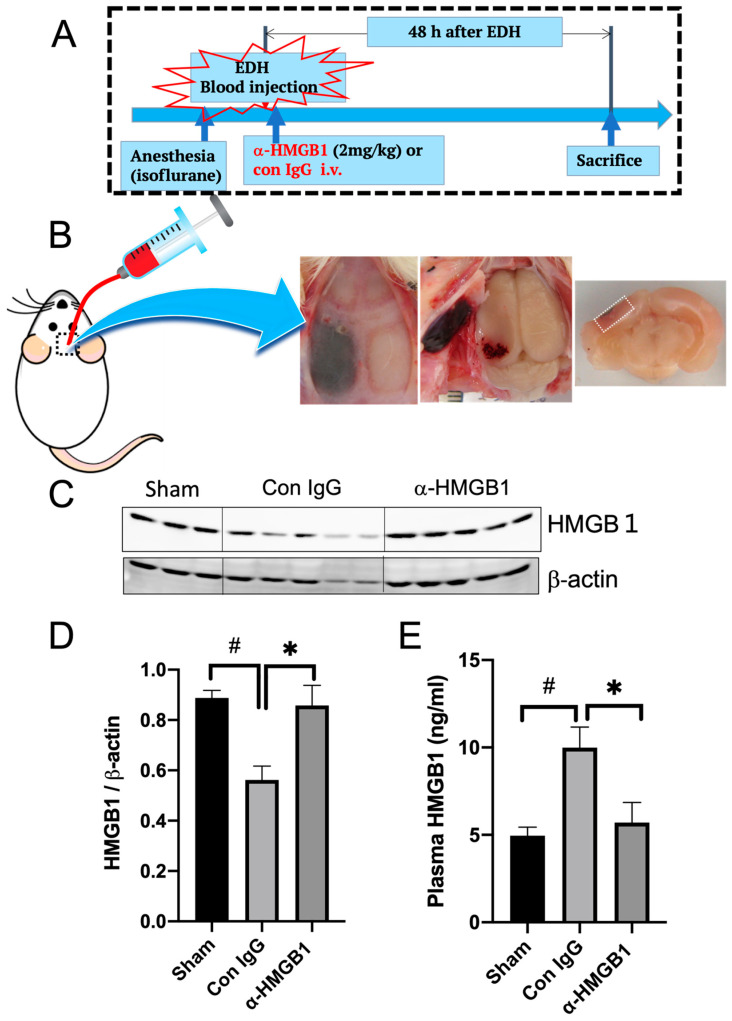
Experimental protocol and changes in HMGB1 levels in the brain and plasma 48 h after the EDH. (**A**) Experimental protocol of the EDH. (**B**) Typical appearance picture of the EDH. The brain samples were collected 48 h after the induction of the EDH from the dotted area in (**B**) and were used for western blotting for HMGB1. (**C**) The representative results of western blotting are shown. A decrease in HMGB1 levels in the ipsilateral cortex (peri-hematoma) in the control IgG group is shown. (**D**) Quantitative analyses of western blotting results were performed using NIH Image J software V.1.53, and the results are expressed as mean ± SE of 3–5 rats per group. Differences were considered significant at ^#^ *p* < 0.05 compared to the sham group. * *p* < 0.05 compared to the control IgG-treated group. (**E**) Plasma levels of HMGB1 in rats with an EDH were determined by ELISA. Blood samples were collected 48 h after the induction of hemorrhage. The results are expressed as mean ± SE of 3–5 rats per group. ^#^ *p* < 0.05 compared to the sham group. * *p* < 0.05 compared to the control IgG-treated group.

The EDH was induced by a whole blood injection (0.2 mL) into the epidural space of the temporal lobe of rats, as shown in Figure 1B. The anti-HMGB1 mAb or control IgG was administered to rats 10 min after blood injection. Then, 48 h after blood injection, all the rats were sacrificed, and the EDH was confirmed in all cases, with no difference in hematoma size between the two groups. We analyzed the HMGB1 levels in the indicated brain tissue beneath the hematoma using western blotting with beta-actin serving as the reference (Figure 1C). The HMGB1 levels in the control rats with the EDH were significantly lower than those in the sham rats. However, treatment with the anti-HMGB1 mAb prevented the reduction in brain HMGB1 levels and maintained the HMGB1 levels at the sham control levels (Figure 1D). In contrast, the plasma levels of HMGB1 in the control rats with the EDH were two-fold higher than those in the sham rats, which was suppressed by the anti-HMGB1 treatment (Figure 1E).

### 2.2. Effects of the Anti-HMGB1 mAb on the Expression of Inflammation-Related Molecules and Apoptosis after the EDH

**Figure 2:**
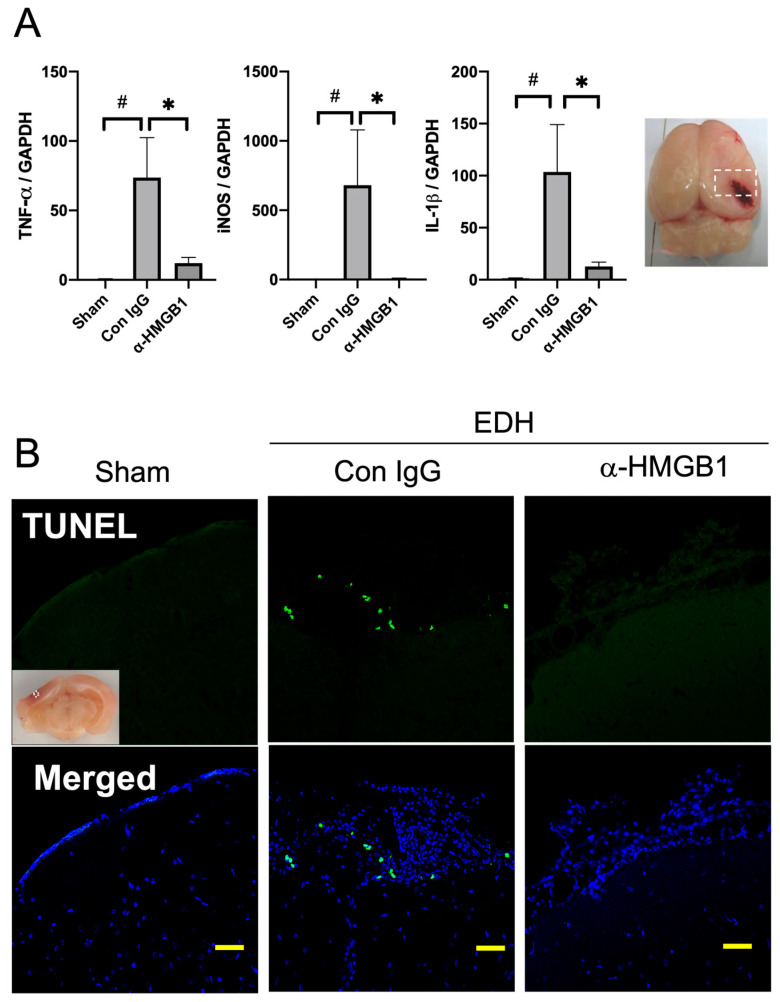
Effects of the anti-HMGB1 mAb on the expression of inflammation-related molecules and apoptosis 48 h after the EDH. (**A**) The mRNA expression of TNF-α, iNOS, and IL-1β was measured by quantitative real-time polymerase chain reaction in the cerebral cortex (peri-hematoma) 48 h after the EDH. The primer information refers to previous research [12]. The results are expressed as mean ± SE of 5 rats (**A**). ^#^ *p* < 0.05 compared to the sham group. * *p* < 0.05 and compared to the control IgG-treated group. (**B**) TUNEL staining was performed to reveal apoptotic cells in the ipsilateral cerebral cortex in each group. Scale bar: 50 μm.

The expression of inflammation-related molecules, including TNF-α, IL-1β, and inducible nitric oxide synthase (iNOS) was determined in the brain area beneath the EDH. The mRNA expression of all three molecules in the control hematoma group was much higher than that in the sham control. The treatment with the anti-HMGB1 mAb inhibited each expression by more than 85% (Figure 2A). Additionally, TUNEL staining was performed to detect apoptotic cells (Figure 2B). In the group where the EDH was treated with control IgG, numerous TUNEL-positive cells were observed within a field, whereas few positive cells were detected in the group treated with the anti-HMGB1 mAb (Figure 2B).

### 2.3. Changes in the Brain Levels of Peroxiredoxins and Heat Shock Proteins in the Brain after the EDH

**Figure 3:**
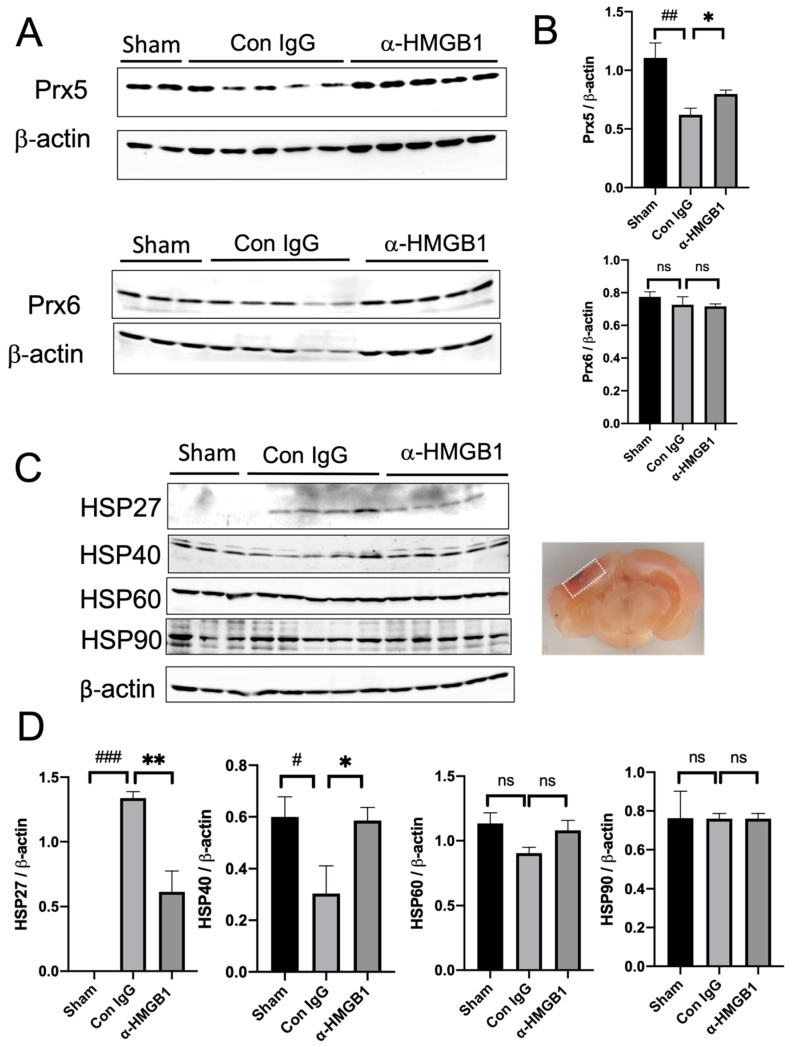
Changes in the brain levels of Prxs and heat shock proteins (HSPs) in the brain after the EDH. (**A**) Prx5 and Prx6 levels in the ipsilateral cerebral cortex (beneath hematoma) were determined 48 h after the EDH by western blotting. (**B**) The representative results of western blotting are shown. β-actin was used as a reference. Quantitative analyses on western blotting results were performed using NIH Image J software. The results are expressed as mean ± SE of 3–5 rats. ^##^ *p* < 0.01 compared to the sham group. * *p* < 0.05 compared to the control IgG-treated group. ns means no significant difference. (**C**) HSP levels (HSP27, HSP40, HSP60, and HSP90) were determined by western blotting in the ipsilateral cortex (beneath hematoma) 48 h after the EDH. The representative results of western blotting are shown. (**D**) Quantitative analyses on western blotting results were performed using NIH Image J software. The results are expressed as mean ± SE of 3–5 rats. ^###^ *p* < 0.001 and ^#^ *p* < 0.05 compared to the sham group. ** *p* < 0.01 and * *p* < 0.05 compared to the control IgG-treated group. ns means no significant difference.

The protein levels of other DAMPs, including peroxiredoxin 5/6 (Prx5/6) [13] and heat shock proteins (HSP27, HSP40, HSP60, and HSP90) were assessed in the brain area beneath the EDH 48 h after the EDH. The levels of Prx5 were found to be reduced in the EDH rats (Figure 3A,B), which was significantly inhibited by the treatment with the anti-HMGB1 mAb. On the other hand, the protein levels of Prx6 did not change in the EDH rats. Among the HSPs, inducible HSP27 protein levels were markedly increased 48 h after the EDH, which was significantly inhibited by the anti-HMGB1 mAb. Meanwhile, HSP40 levels were reduced, which were restored by anti-HMGB1 mAb treatment. No significant changes were observed in the HSP60 and HSP90 levels at this time point (Figure 3C,D).

### 2.4. Expression of AQP4, PAR1, PAI-1, and MSR1 after the EDH and Effects of Anti-HMGB1 Treatment

**Figure 4:**
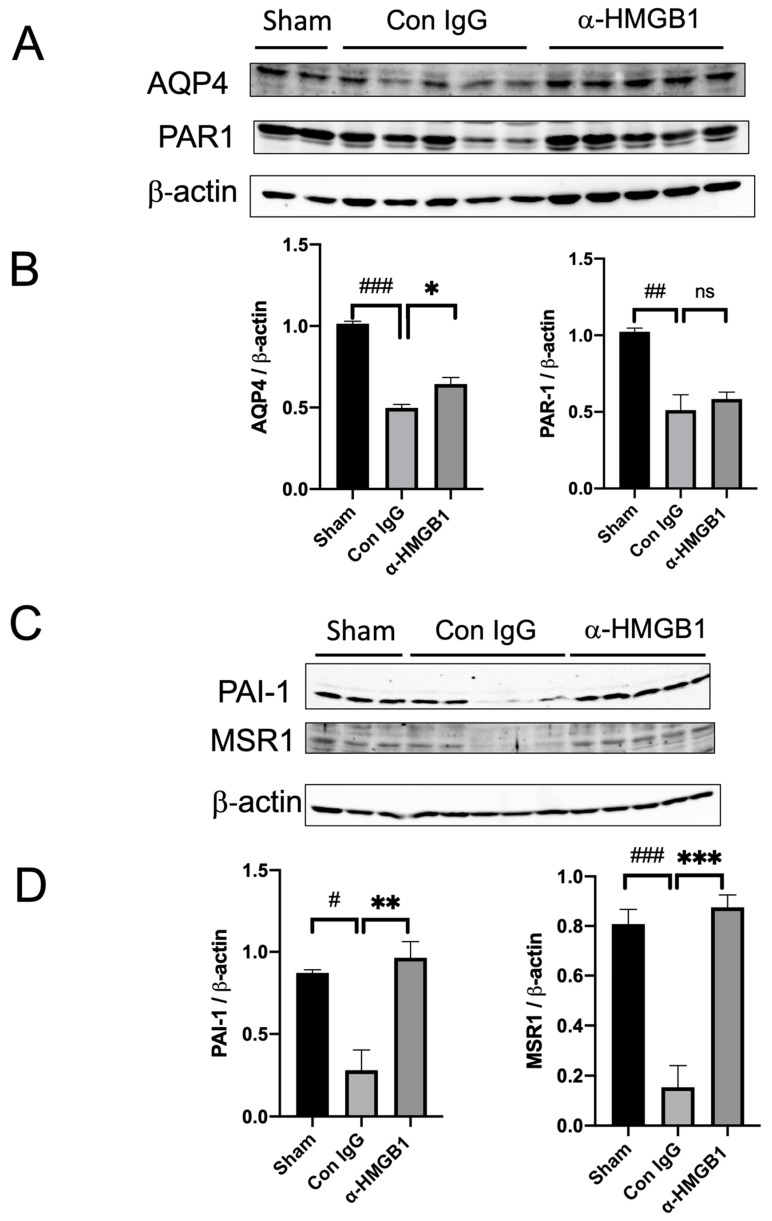
Expression of AQP4, PAR1, PAI-1, and MSR1 after the EDH and effects of anti-HMGB1 treatment. (**A**) The protein levels of AQP4 and PAR-1 in the ipsilateral cerebral cortex (beneath the hematoma) were determined by western blotting 48 h after the EDH. The representative results of western blotting are shown. (**B**) The quantitative results are expressed as mean ± SE of 3–5 rats. The protein levels of AQP4 and PAR-1 in the ipsilateral cerebral cortex (beneath the hematoma) were determined by western blotting 48 h after the EDH. ^##^ *p* < 0.01, and ^###^ *p* < 0.001 compared to the sham group. * *p* < 0.05 compared to the control IgG-treated group. ns means no significant difference. (**C**) The protein levels of PAI-1 and MSR-1 in the ipsilateral cerebral cortex (beneath the hematoma) were determined by western blotting 48 h after the EDH. The representative results of western blotting are shown. (**D**) The quantitative results are expressed as mean ± SE of 3–5 rats. The protein levels of PAI-1 and MSR1 in the ipsilateral cerebral cortex (beneath the hematoma) were determined by western blotting 48 h after the EDH. ^#^ *p* < 0.05, and ^###^ *p* < 0.001 compared to the sham group. ** *p* < 0.01, and *** *p* < 0.01 compared to the control IgG-treated group.

Following the EDH, the levels of aquaporin-4 (AQP4), a key water channel protein involved in brain edema, and thrombin receptor PAR-1, which is implicated in coagulation and inflammation, were both reduced by 50%. Anti-HMGB1 treatment partially reversed the decrease in AQP4 levels, suggesting its potential role in mitigating brain edema, but did not significantly affect the reduction in PAR-1 levels (Figure 4A,B). Additionally, the protein levels of plasminogen activator inhibitor-1 (PAI-1), a regulator of fibrinolysis, and macrophage scavenger receptor 1 (MSR1), which is involved in the clearance of cellular debris, were markedly suppressed 48 h after the EDH. Anti-HMGB1 treatment completely reversed the suppression of PAI-1 and MSR1, indicating its efficacy in restoring these proteins’ expression and potentially enhancing the brain’s recovery process (Figure 4C,D)

### 2.5. Effects of the Anti-HMGB1 mAb on Stimulant-Induced HMGB1 Translocation in Neuroblastoma Cells and Vascular Endothelial Cells

**Figure 5:**
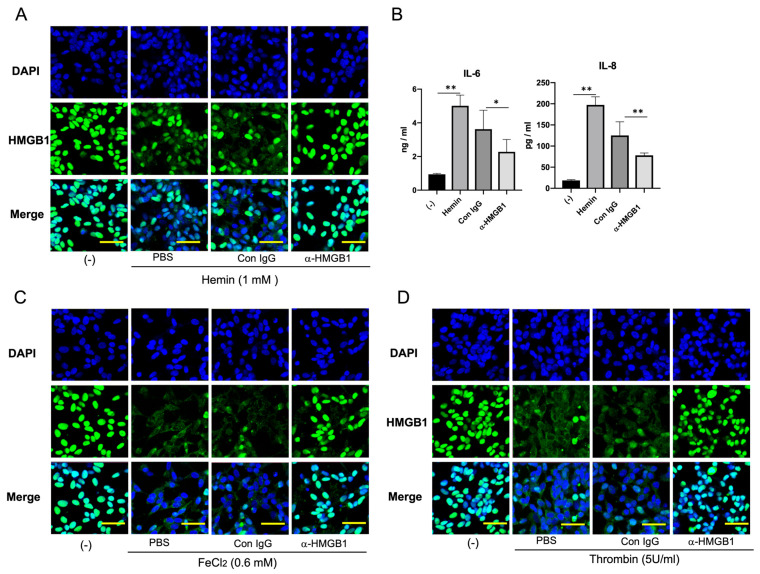
Effects of the anti-HMGB1 mAb on stimulant-induced HMGB1 translocation in neuroblastoma cells in culture. (**A**) SH-SY5Y neuroblastoma cells were stimulated with hemin (1 mM) for 24 h, and the translocation of HMGB1 was observed immunocytochemically. The anti-HMGB1 mAb (10 μg/mL) or anti-KLH mAb (control) was added to the media immediately before the start of incubation. DAPI was used for nuclear staining. Scale bar: 20 μm. (**B**) IL-6 and IL-8 levels in the media stimulated with hemin (0.6 mM) were determined 24 h after stimulation in the presence (10 μg/mL) or absence of the anti-HMGB1 mAb. The results are expressed as mean ± SE of triplicate experiments. * *p* < 0.05 and ** *p* < 0.01 compared to the non-stimulated group or to the control IgG-treated group, as shown by the lines in the figure. (**C**) SH-SY5Y neuroblastoma cells were stimulated with FeCl_2_ (0.6 mM) for 24 h in the presence or absence of the anti-HMGB1 mAb (10 μg/mL), and the translocation of HMGB1 was observed immunocytochemically. Scale bar: 20 μm. (**D**) SH-SY5Y neuroblastoma cells were stimulated with thrombin (5 U/mL) for 24 h in the presence or absence of the anti-HMGB1 mAb (10 μg/mL), and the translocation of HMGB1 was observed immunocytochemically. Scale bar: 20 μm.

**Figure 6:**
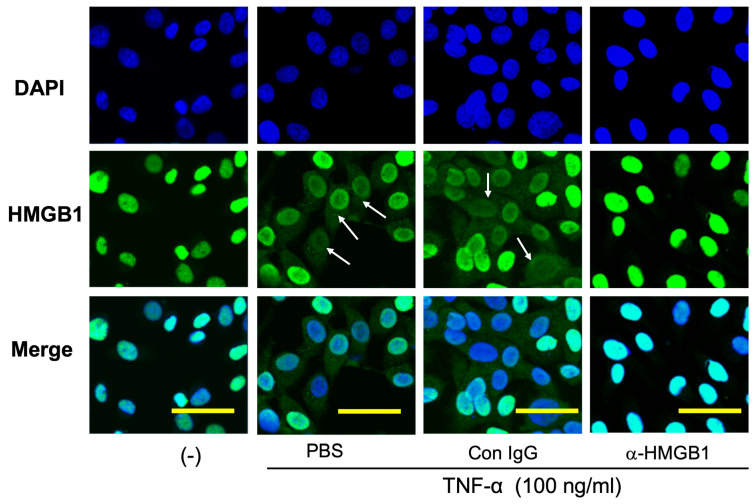
Effects of the anti-HMGB1 mAb on TNF-α-induced HMGB1 translocation in vascular endothelial cells in culture. EA.hy 926 vascular endothelial cells were stimulated with TNF (100 ng/mL) in the presence or absence of the anti-HMGB1 mAb (10 μg/mL) for 6 h, and the translocation of HMGB1 was observed immunocytochemically. DAPI was used for nuclear staining.

In the in vitro experiments, we observed that hemin (1 mM), ferrous iron (0.6 mM), and thrombin (5 U/mL) induced the translocation of HMGB1 from the nuclei to the cytosolic compartment in SH-SY5Y, a neuronal cell line, when determined 24 h after the start of incubation (Figure 5A,C,D). The addition of the anti-HMGB1 mAb (10 μg/mL) to the incubation medium significantly inhibited the translocation of HMGB1 under all three stimulants, whereas the control mAb (10 μg/mL) had no effect on HMGB1 translocation. Figure 5B shows that the addition of the anti-HMGB1 mAb (10 μg/mL), but not the control mAb, significantly reduced the production of IL-6 and IL-8 induced by hemin (0.6 mM). In EA.hy 926 vascular endothelial cells, similar to the observations for SH-SY5Y cells, TNF-α (100 ng/mL) induced the translocation of HMGB1. The translocation of HMGB1 was markedly inhibited by the addition of the anti-HMGB1 mAb (10 μg/mL) but not by the control mAb (Figure 6).

## 3. Discussion

In the present study, we demonstrated that anti-HMGB1 mAb treatment significantly inhibited the inflammatory responses in the brain region beneath the EDH. The EDH was induced by the direct injection of autologous blood into the epidural space, followed by the systemic injection of the anti-HMGB1 mAb. The inhibitory effects of anti-HMGB1 mAb treatment can be attributed to the suppression of secondary responses following the EDH. Notably, the induction of the mRNA expression of TNF-α, IL-1β, and iNOS was strongly inhibited by anti-HMGB1 treatment, indicating the effective suppression of key inflammatory cytokines. In addition, anti-HMGB1 mAb treatment efficiently inhibited the release of HMGB1 in the affected brain areas. These anti-inflammatory effects, including the reduction of cytokine production and HMGB1 release, align with observations from different brain injuries induced by ischemia/reperfusion [9,10], hemorrhage [12], trauma [11], and epilepsy [4,14] in various studies. Under the conditions listed above, there was marked translocation and release of HMGB1, mainly from neurons in the affected areas, leading to the reduction in brain HMGB1 content and a concurrent increase in plasma [4,9,11]. A similar pattern of HMGB1 dynamics was observed in the present EDH model, suggesting that the anti-HMGB1 mAb may be effective in mitigating secondary inflammatory processes across various types of brain injuries.

In acute EDHs, brain injuries manifest predominantly through two mechanisms: compression-induced trauma and ischemia in the relevant areas and injuries induced by clot components such as thrombin and heme/iron. In our previous study [11], we showed that fluid percussion-induced brain trauma in rats leads to remarkable HMGB1 translocation and release, mainly from neurons in the affected areas, within 24–48 h after the injury. This released HMGB1, in turn, affected the BBB structural integrity, thereby disrupting its function and contributing to brain edema [5,9,11], along with the activation of microglia. The resulting brain edema further facilitates brain ischemia, creating a feedback loop that enhances the acute inflammatory response in the brain [5]. Thus, HMGB1 emerges as both a mediator and an enhancer in the vicious cycle of the acute phase of brain inflammatory responses [5].

In contrast to the decreased levels of HMGB1 in the brain area beneath the EDH 48 h after insult, the plasma levels of HMGB1 were elevated at the same time point. This elevation was almost completely reversed by the treatment with the anti-HMGB1 mAb. The relationship between the decreased HMGB1 levels in the affected brain area and the increased plasma levels strongly suggests that HMGB1 released from affected brain areas contributes to the elevation of plasma HMGB1 levels, as indicated in the different brain injury models [4,9,11,12,15]. Clinical studies have shown that isolated acute brain injuries can cause the dysfunction of peripheral extracranial organs, especially the lungs [16], and HMGB1 released from affected brain tissues into circulation during different brain injuries possibly contributes to the amplification of inflammatory responses in the target organs [17,18].

In addition to cytokine production suppressing the effects of the anti-HMGB1 mAb, we demonstrated that mAb inhibited the dynamics of Prx5, another DAMP [13], and chaperone molecules HSP27 and HSP40. These molecules have been suggested to contribute to cellular stress responses and inflammation modulation. Thus, the acute treatment with anti-HMGB1 effectively suppressed the release and function of these critical inflammatory mediators, reducing both cellular stress and systemic inflammatory responses. This broad-spectrum suppression may contribute significantly to ameliorating the secondary damage typically observed in acute brain injury scenarios.

In previous studies, we demonstrated that treatment with the anti-HMGB1 mAb effectively inhibited the translocation and release of HMGB1 from neurons in the affected brain areas [9,10,11,12]. It appears that BBB disruption is, at least partially, mediated by HMGB1 during brain insults [9,11]. The systemic administration of the anti-HMGB1 mAb probably binds to released HMGB1 around the BBB and inhibits its disruption. This inhibition reduces BBB permeability and maintains its function, leading to a decrease in inflammatory responses within the brain parenchyma. Consequently, the inflammatory processes such as cytokine production and microglia activation are closely linked to BBB integrity. This highlights a reciprocal relationship where disruption of the BBB and inflammatory responses influence each other, contributing to the progression of brain damage [5].

In the present study, we showed that both thrombin and heme/ferrous ions induced the translocation of HMGB1 in SH-SY5Y neuronal cells in vitro. During hematoma formation, thrombin production represents the final phase of the coagulation cascade. Subsequently, thrombin that has extravasated from the hematoma infiltrates the adjacent, compressed regions of the cerebral cortex, which are rich in neurons. This infiltration triggers the release of HMGB1 from these neurons. Thrombin is also known to exert deleterious effects on neurons, astrocytes, microglia, and vascular endothelial cells under various conditions [19]. Therefore, thrombin may enhance the inflammatory responses by facilitating HMGB1 release. As a result, the neutralization of HMGB1 by anti-HMGB1 may block the synergistic pathway between thrombin and HMGB1, attenuating the inflammatory cascade. Moreover, hemoglobin degradation products, specifically heme and ferrous ions, are known to generate ROS. This ROS production produces oxidative stress in neurons, facilitating inflammatory responses. Our findings indicate that the translocation of HMGB induced by thrombin and heme/ferrous ions can be effectively inhibited by the addition of anti-HMGB1 to the culture medium. This strongly suggests a potential rapid positive feedback loop in the HMGB1 release. Similar inhibition of HMGB1 release by anti-HMGB1 monoclonal antibodies was observed in cultures of vascular endothelial cells stimulated with LPS or TNF-α.

AQP4 is mainly expressed in astrocytic processes surrounding the capillaries in the brain [20] and has been implicated in the biphasic regulation of brain edema [21,22]. In our previous paper [12], we investigated the localization of AQP4 by immunostaining in the hematoma-surrounding area of an intracerebral hemorrhage model. AQP4 expression surrounding the capillaries was significantly increased 24 h after hemorrhage. However, anti-HMGB1 mAb treatment maintained AQP4 expression at levels comparable to the sham group. In this study, we observed that AQP4 expression completely disappeared 48 h after the EDH. This could be because, following the establishment of brain edema due to BBB disruption (vasogenic edema), AQP4 deletion can delay the edema resolution process. This suggests that the slight elevation in AQP4 levels, induced by the anti-HMGB1 mAb in the present study, may facilitate the recovery phase of brain edema.

PAI-1 is a major regulator of the t-PA/plasmin system in plasma, controlling fibrinolysis. Moreover, the balance between PAI-1 and serine proteinases is critical for the repair of damaged brain tissue [23]. Buisson et al. have reported that the up-regulation of PAI-1 in astrocytes confers protection against excitotoxic neuronal death, suggesting that PAI-1 expression could represent a novel therapeutic target for stroke [23]. In our study, we observed a marked decrease in PAI-1 levels in the brain area beneath the EDH in rats, indicating a disruption in the balance between PAI-1 and serine proteinases. This imbalance was effectively reversed by the anti-HMGB1 mAb. Additionally, MSR1 was reported to play an important role in the clearance of plural DAMPs such as HMGB1, S100A8/A9, and peroxiredoxins after ischemic brain injury [24]. In our current study, we observed that the administration of anti-HMGB1 monoclonal antibodies reversed the suppression of MSR1 expression observed under EDH conditions. This restoration of MSR1 levels potentially facilitates the repair process after injury by enhancing the clearance of DAMPs.

Under normal conditions, HSP27 is seldom expressed in brain tissues but is predominantly induced in astrocytes within ischemic brain regions [25]. In our study, the induction of HSP27 in the brain area beneath the EDH was significantly elevated compared to the sham controls, indicating a persistent stress response characterized by the solubilization of misfolded proteins in astrocytes. Furthermore, a 50% reduction in HSP27 induction by anti-HMGB1 treatment supports the notion that the neutralization of HMGB1 could indirectly produce beneficial effects on astrocytes.

Proteins in the peroxiredoxin (Prx) family were originally described as intracellular antioxidative enzymes that reduce ROS, such as peroxides, and play neuroprotective roles [26]. Prxs are categorized based on their cysteine residues: Prx6 contains one, while Prx1–Prx5 contains two. However, once released from the necrotic cells, Prxs are considered to lose their antioxidant capacity due to the oxidation of the cysteine residues, rendering the redox cycle inoperable. Conversely, extracellular Prxs may act as DAMPs through the stimulation of TLR-2 and TLR-4, which strongly induce IL-23 expression in bone marrow-derived dendritic cells (BMDCs) [13]. In an MCAO mouse model, it has been demonstrated that Prxs are released from necrotic cells into the extracellular fluid, where they play a very important role in triggering brain inflammation. Among the Prx family, Prx5 emerges as the most potent inducer of interleukin-23 (IL-23), highlighting its significant role in mediating inflammatory responses [13]. In the present EDH model, a 50% decrease in Prx5 in the affected area was observed, suggesting a substantial extracellular space. Notably, the application of anti-HMGB1 mAb therapy significantly countered this reduction, implying that the treatment with the mAb suppresses necrosis in affected cells.

In vitro experiments showed that thrombin and hemoglobin degradation products, specifically heme and ferrous iron, induced the HMGB1 translocation in neuronal cell line SH-SY5Y. A similar pattern of HMGB1 translocation was also observed in vascular endothelial cell cultures stimulated by TNF-α. The effective inhibition of this translocation in both cell types by the neutralization of endogenously released HMGB1 with the anti-HMGB1 monoclonal antibody suggests the existence of a positive feedback loop. In other words, the translocation and release of HMGB1 were accelerated by extracellular HMGB1 acting in autocrine and paracrine manners. The suppression of cytokine release from these cells by the anti-HMGB1 mAb supports its potential as a potent inhibitor of inflammatory responses in vivo [18,27]. However, the mechanism for such positive feedback remains to be determined [5].

In conclusion, our findings demonstrate the beneficial effects of the systemic injection of the anti-HMGB1 mAb in mitigating brain inflammation and injury associated with the EDH. These results suggest that anti-HMGB1 mAb therapy may provide an additional treatment option for the EDH, complementing traditional neurosurgical hematoma removal.

## 4. Materials and Methods

### 4.1. Animals and Treatment Groups

Adult male Wistar rats (weight 300–350 g) were used in this study. All experimental procedures were conducted in accordance with the Okayama University Guidelines for Animal Experiments and were approved by the university’s Committee on Animal Experiments (approval no. OKU-2020189). All rats were randomly allocated into the following 3 groups: (1) the anti-HMGB1 mAb group (α-HMGB1), in which the EDH was induced by the injection of 300 ul of autologous blood without anticoagulant and then the anti-HMGB1 mAb (which was made in the lab and recognizes the C-terminal sequence of the HMGB1 molecule (EEEDDDDE) [10], (#10-22, IgG2a subclass, and 2 mg/kg)) was administered intravenously within 10 min and 6 h after EDH induction; (2) the class-matched control mAb group (Con IgG), in which the EDH was induced and then the class-matched control mAb (anti-Keyhole Limpet hemocyanin IgG2a, 2 mg/kg) was administered intravenously; and (3) the sham group, which underwent the same procedures as the other groups, except the injection of autologous blood, and was treated with the same volume of saline.

### 4.2. Surgical Induction of the EDH

EDH model was established according to the surgical procedure of Eijkenboom et al. [28] with some modifications. In short, under anesthesia with 3.0% isoflurane (#v002139, VTRS, Tokyo, Japan) in a mixture of 50% oxygen and 50% nitrous oxide gas, burr holes (diameter of 3 mm) were drilled into the skull above the cortex (coordinates: 3.0 mm lateral and 1.0 mm posterior from bregma). Subsequently, autologous nonheparinized blood was obtained from the tail and was immediately injected via a specially designed plastic cannula (inner diameter: 0.4 mm and outer diameter: 0.8 mm) into the epidural space (1 mm under skull surface) at a rate of 50 μL/min (total volume: 300 μL/rat). Ten minutes later, the plastic cannula was cut off flush with the skull, and the burr hole was filled with bone wax, leaving the tip of the plastic cannula in place.

### 4.3. Determination of HMGB1 by ELISA

Plasma levels of HMGB1 were determined by an enzyme-linked immunosorbent assay (ELISA) (# 326078738, Shinotest, Tokyo, Japan), as described previously [12].

### 4.4. Histological Study

For the histological study, the rats were anesthetized with an i.p. injection of sodium pentobarbital (50 mg/kg) and perfused through the left ventricle with saline, followed by 4% paraformaldehyde (163-20145, Wako Pure Chemical Industry, Osaka, Japan). Paraffin-embedded brain sections (5-μm thick) were used for the terminal deoxynucleotidyl transferase-mediated dUTP nick end labeling (TUNEL) staining or immunofluorescence study. For the TUNEL assay (MK500, Takara, Shiga, Japan), paraffin-embedded brain sections were stained following the manufacturer’s guidelines. Finally, sections were mounted using the Mounting Medium with DAPI and observed under a confocal imaging system (LSM 780, ZEISS, Oberkochen, Germany).

### 4.5. Cell Culture

The neuroblastoma cell line SH-SY5Y was generously gifted by Dr. Sogawa (Department of Dental Pharmacology, Okayama, Japan). SH-SY5Y cells were cultured in Dulbecco’s modified Eagle medium (DMEM, #D6546, Sigma, St. Louis, MO, USA) supplemented with 5% L-glutamine (#G7513, Sigma, St. Louis, MO, USA), 10% fetal bovine serum (Gibco, Grand Island, NY, USA), and 10% penicillin/streptomycin (#15140122, Gibco, Tokyo, Japan) at 37 °C in a 5% CO_2_ atmosphere. The EA.hy926 cell line (ATCC Cat# CRL-2922, RRID: CVCL_3901), a hybridoma of a human umbilical vein endothelial cell (HUVEC), and adenocarcinoma human alveolar basal epithelial cell A549 were cultured as described above. After reaching confluence, the EA.hy926 cells were detached from culture flasks as described previously [29]. These cells were utilized for experiments between their third and sixth passages.

### 4.6. Immunocytochemistry

SH-SY5Y cells pretreated with 10 ug/mL of the anti-HMGB1 mAb or Control IgG for 1 h (h) before being stimulated with hemin (51280, Sigma, Tokyo, Japan) (1 mM), FeCl_2_ (372870, Sigma, Tokyo, Japan) (0.6 mM), or thrombin (B352, Mochida, Tokyo, Japan) (5 U/mL). Then, 24 h after stimulation, the cells were fixed with 4% paraformaldehyde and blocked with 10% bovine serum albumin (BSA). Afterward, the cells were stained by the anti-HMGB1 Ab (ab18256, Sigma, Tokyo, Japan) for 1 h at 37 °C, followed by Alexa Fluor 488-labeled anti-rabbit IgG. The cell nuclei were stained with DAPI for 5 min and then observed using a confocal microscope (LSM 780, ZEISS, Oberkochen, Germany). The EA.hy 926 cells were prepared as described previously [15]. The EA.hy 926 cells were also pretreated with 10 ug/mL of the anti-HMGB1 mAb or control IgG for 30 min before being stimulated with Tumor Necrosis Factor-alpha (TNF-α) (H8916, Sigma, Tokyo, Japan) (100 ng/mL) for 6 h. The immunostaining for HMGB1 was conducted as described above.

### 4.7. Western Blotting

Brain samples (9 mm^2^) were collected from both the hematoma and contralateral sides 48 h after injury and homogenized in N-PER lysis buffer (#87792, Thermos Scientific, NY, USA) with protease inhibitors (p8340, sigma, St. Louis, MO, USA). The lysates were centrifuged, and the supernatants were collected for protein quantification using the BCA assay. Equal amounts of protein (10 μg) were resolved on 10% SDS-PAGE gels and transferred to nitrocellulose membranes. The membranes were blocked with 5% non-fat dry milk and then incubated overnight at 4 °C with primary antibodies against HMGB1 (ab18256, Abcam, Waltham, MA, USA), Heat Shock Protein 27 (Hsp27) (ab2790, Abcam, Waltham, MA, USA), Hsp40 (ab69402, Abcam, Waltham, MA, USA), Hsp60 (ab46798, Abcam, Waltham, MA, USA), Hsp90 (ab13492, Abcam, Waltham, MA, USA), Peroxiredoxin 5 (Prx5) (ab180587, Abcam, Waltham, MA, USA), Prx6 (ab195045, Abcam, Waltham, MA, USA), Aquaporin-4 (AQP4) (ab9512, Abcam, Waltham, MA, USA), Protease-Activated Receptor 1 (PAR1) (PA5-116040, Invitrogen, Carlsbad, CA, USA), and Plasminogen Activator Inhibitor-1 (PAI-1) (ab66705, Abcam, Waltham, MA, USA). β-actin (sc-47778, Santa Cruz, CA, USA) was used as a reference protein. After washing, the membranes were incubated with HRP-conjugated secondary antibodies (sc-2005 or sc-2004, Santa Cruz, CA, USA), developed with Enhanced Chemiluminescence, and the signals were detected.

### 4.8. Cytometric Bead Array (CBA) Measurement

The SH-SY5Y cells were subcultured in 6-well plates (5 × 10^5^ cells/mL). After 24 h, the cells were treated with 0.6 mM hemin in the presence or absence of an anti-HMGB1 monoclonal antibody (mAb, 10 μg/mL) for an additional 24 h. Then, the cell-free supernatant was collected, and Interleukin-6 (IL-6) and IL-8 were measured by CBA kits (#551811, BD Biosciences, Franklin Lakes, NJ, USA) following the manufacturer’s instructions. Briefly, the supernatants were incubated with capture beads and detection antibodies, and the samples were analyzed using a flow cytometer (MACSQuant, Burladingen, Germany). The data were acquired and analyzed using the MACSQuant software V3.0.

### 4.9. RT-PCR

The EA.hy 926 cells were pretreated with the anti-HMGB1 mAb or anti-KLH mAb (1 ug/mL) for 30 min before the stimulation with TNF-α (100 ng/mL) for 8 h. The EA.hy 926 cells were harvested, and mRNA was extracted using an RNeasy (Qiagen, Hilden, Germany). Complementary DNA was synthesized with a Takara RNA PCR kit ver. 3.0 (RR019A, Takara Bio, Nagahama, Japan), and RT-PCR were performed with a Light Cycler (Roche, Basel, Switzerland) according to the manufacturer’s instructions. The primers (β-actin: Forward 5′-AGCGGGAAATCGTGCGTG-3′ and Reverse 5′-CAGGGTACATGGTGGTGCC-3′; IL-6: Forward 5′-GAACTCCTTCTCCACAAGCGCCTT-3′ and Reverse5′-CAAAAGACCAGTGATGATTTTC ACCAGG-3′; and IL-8: Forward 5′-ATGACTTCCAAGCTGGCCGTGGCT-3′ and Reverse 5′-TCTCAGCCCTCTTCAAAAACTTCTC-3′) were used to amplify specific cDNA fragments. The β-actin expression was used to normalize the cDNA levels.

### 4.10. Statistical Analysis

In vitro data are presented as the mean ± SE of at least 3 separate experiments. All values were analyzed using an analysis of variance (ANOVA) followed by Bonferroni’s test or post-hoc Fisher’s test using Prism 9 software (Boston, MA, USA). *p*-values less than 0.05 were considered to be statistically significant.

## Data Availability

If readers need any raw data, please contact the corresponding author.
